# Psychological resilience partially mediates the relationship between negative life events and suicidal ideation in Chinese adolescents

**DOI:** 10.3389/fpsyt.2026.1770147

**Published:** 2026-08-20

**Authors:** Wanmeng Wu, Lili Liu, Zahari Ishak, Zhigang Wang, Jiaqi Sun, Zhuoyu Han

**Affiliations:** 1 School of International Education and Exchange, College of Science and Technology Changchun, Changchun, China; 2 School of Education, Changchun Normal University, Changchun, China; 3 Faculty of Business, Information & Human Sciences (FBIHS), Kuala Lumpur University of Science & Technology (KLUST), Selangor Darul Ehsan, Malaysia; 4 Student affairs office, College of Science and Technology Changchun, Changchun, China

**Keywords:** adolescents, mediating effect, negative life events, psychological resilience, suicidal ideation

## Abstract

**Background:**

Suicidal ideation is a significant public health concern among adolescents globally, with negative life events and psychological resilience identified as key risk and protective factors, respectively.

**Objective:**

To explore the mediating effect of psychological resilience between negative life events and suicidal ideation in adolescents, and to analyze differences in related variables across demographic characteristics.

**Methods:**

This cross-sectional study analyzed publicly available data from the Global School-based Student Health Survey (GSHS) conducted in China, including adolescents aged 13–17 years after data cleaning. Negative life events, psychological resilience proxies (e.g., parental support, peer connectedness), and suicidal ideation were measured using standardized items in the GSHS questionnaire. Data analysis was conducted using structural equation modeling, with the mediating effect tested using the Bootstrap method.

**Results:**

The mean composite score for negative life events was 8.2 ± 4.8, for psychological resilience was 14.3 ± 3.6, and for suicidal ideation was 0.8 ± 1.2. Negative life events were significantly positively correlated with suicidal ideation (r = 0.456, p< 0.001), while psychological resilience was significantly negatively correlated with both negative life events (r = -0.512, p< 0.001) and suicidal ideation (r = -0.623, p< 0.001). Suicidal ideation showed significant differences by gender, age group, and parental support (P< 0.05). Psychological resilience partially mediated the relationship between negative life events and suicidal ideation, with a mediation effect value of 0.069, accounting for 17.56% of the total effect (95% CI: 0.036—0.527, excluding 0).

**Conclusion:**

Negative life events were directly and indirectly associated with suicidal ideation among adolescents through psychological resilience. It is recommended to strengthen psychological resilience interventions for adolescents to mitigate the impact of negative events and promote mental health.

## 1 Introduction

Suicidal ideation—defined as thoughts, plans, or wishes to end one’s own life, ranging from passive death wishes to active planning—among adolescents represents a significant global public health concern (1). Approximately 13.6% of adolescents worldwide report suicidal thoughts, with rates in China ranging from 22% to 31% depending on regional and temporal contexts (2–4). As a key precursor to suicidal behavior, understanding the mechanisms underlying suicidal ideation is essential for developing effective prevention strategies.

Negative life events—including interpersonal conflicts, academic pressure, bullying, and family adversity—are consistently identified as primary risk factors for adolescent suicidal ideation (5, 6). Cumulative exposure to such stressors is associated with emotional dysregulation, depression, and increased suicidal ideation, with effects potentially extending into adulthood (7, 8).

The Adverse Childhood Experiences (ACE) framework has established that cumulative exposure to childhood adversities—including abuse, neglect, and household dysfunction—is associated with elevated risks of mental health problems and suicidal behavior across the lifespan (9). ACE research has consistently identified a dose-response relationship between the number of adversities and adverse psychological outcomes, with resilience-related protective factors proposed as a key moderating mechanism. The present study extends this line of inquiry by examining resilience-related resources as a mediating pathway specifically within the adolescent developmental period.

Psychological resilience—defined as the capacity to successfully adapt to adversity through psychological, emotional, and behavioral flexibility—serves as a critical protective factor (10, 11). Resilience encompasses internal resources (e.g., emotional regulation, positive cognition) and external supports (e.g., family, peer relationships), enabling individuals to recover from stress and maintain well-being. Higher resilience is associated with reduced risk of negative mental health outcomes, including suicidal ideation, among adolescents (12). Resilience-based interventions have demonstrated effectiveness in promoting adaptive coping and preventing adverse psychological outcomes.

Previous studies have primarily examined the direct associations between negative life events and suicidal ideation in Western adolescent samples. However, the mediating pathway through which negative life events may affect suicidal ideation via resilience-related protective resources remains underexplored, particularly in Chinese adolescent populations. Understanding whether and to what extent resilience mediates the relationship between adversity and suicidal thoughts is essential for designing targeted interventions. This cross-sectional study examines the mediating role of psychological resilience in the pathway from negative life events to suicidal ideation among Chinese adolescents, using secondary analysis of the Global School-based Student Health Survey (GSHS) data collected in 2003 from four Chinese cities (Beijing, Hangzhou, Wuhan, and Urumqi). By clarifying this mechanism, the study provides empirical evidence to inform resilience-focused prevention and intervention strategies.

While the direct associations between negative life events and suicidal ideation, and between resilience and suicidal ideation, are well established, the specific mediating pathways remain underexplored, particularly in Chinese adolescent populations. Understanding whether and to what extent resilience mediates the relationship between adversity and suicidal thoughts is essential for designing targeted interventions. This study examines the mediating role of psychological resilience in the pathway from negative life events to suicidal ideation among Chinese adolescents, using secondary analysis of a large, nationally representative dataset. By clarifying this mechanism, the study provides empirical evidence to inform resilience-focused prevention and intervention strategies.

## 2 Related work

### 2.1 Review of research on negative life events

Life events are generally considered to be the various social changes that individuals encounter in their daily lives (13). Negative life events are a type of life event and a source of stress, typically referring to a series of changes that individuals face in their daily lives, such as aging, illness, death, and failure. For individuals, negative life events serve as an important social stressor (14).

The scale and nature of negative life events vary, and their subsequent impacts differ accordingly (15). Negative life events experienced during childhood and adolescence are associated with the onset and development of various mental health problems and may also influence emotional and behavioral patterns in adulthood (16, 17).

To quantitatively assess negative life events, researchers have developed various scales, such as the Social Readjustment Rating Scale (SRRS) (18), which evaluates stress intensity from common life events. In China, adapted scales like the Adolescent Self-Rating Life Events Checklist (ASLEC) (19) and the Life Events Scale (20) account for cultural contexts, focusing on academic pressure, interpersonal issues, and family changes. These tools highlight how cumulative negative events predict mental health outcomes, setting the stage for examining protective mechanisms like resilience. While these established scales provide valuable frameworks for understanding negative life events, the present study utilizes standardized items from the GSHS, which capture similar constructs through internationally validated measures suitable for cross-cultural comparisons.

### 2.2 Review of resilience research

The concept of “psychological resilience” originated from the American term “resilience” and gained widespread recognition and in-depth study in academic circles during the 1980s (21). Domestic scholars have translated this term into Chinese based on their research focus, including psychological resilience, psychological elasticity, adversity quotient, and resilience.

Through the relentless efforts of researchers, the concept evolved from an initial vague notion to a theoretical model. In the 1980s, Garmezy and colleagues categorized resilience into compensatory, challenge, and protective factor models, laying the foundation for subsequent theoretical development (22). Kumpfer later integrated and extended existing theories to propose the Kumpfer Resilience Framework, which organizes resilience factors across multiple ecological levels (23). Mandleco and Peery further developed a systemic framework of children’s psychological resilience encompassing internal personal factors, psychological state factors, and external environmental factors (24). This framework is primarily divided into three parts: personal internal factors, psychological state factors, and external environmental factors. These factors interact with one another, and factors within each category also influence one another. In the 21st century, research on psychological resilience has become more diverse. Nesbitt et al. expanded and refined the theoretical framework of psychological resilience, introducing a process-oriented model (25). This model focuses on how individuals maintain a stable state, viewing the individual’s mind-body balance as the product of the interaction between protective factors and negative experiences. When faced with numerous threatening factors, protective factors can help individuals regain balance.

Across developmental psychology, clinical psychology, and stress-adaptation theory, resilience is consistently conceptualized as a dynamic system that regulates individuals’ emotional and cognitive responses when encountering stressors. Classic stress theories, such as the stress-response model (26) and contemporary affect-regulation frameworks, propose that negative life events activate physiological and psychological stress pathways, increasing vulnerability to internalizing symptoms, including suicidal thoughts (26).

Resilience theory further clarifies that protective factors-–such as parental support, peer connectedness, and school belonging-–buffer the negative effects of adverse events by enhancing coping flexibility, emotional regulation, and meaning-making processes (27). Mineva (28) emphasize that resilience functions as an affect-regulation mechanism that reduces the intensity and duration of stress-induced negative affect. Empirical studies among adolescents corroborate this pathway, demonstrating that lower resilience is associated with impaired emotion regulation, heightened depressive symptoms, and elevated suicidal ideation (29). Therefore, existing theory supports a mediating pathway in which negative life events are associated with lower resilience, which in turn is associated with a higher likelihood of suicidal ideation.

## 3 Methodology

### 3.1 Research hypotheses

H1: Levels of negative life events, suicidal ideation, and psychological resilience among adolescents differ significantly by demographic characteristics, with female adolescents, younger adolescents, and those with lower parental support reporting higher suicidal ideation.

H2: Negative life events are positively correlated with suicidal ideation and negatively correlated with psychological resilience; psychological resilience is negatively correlated with suicidal ideation among adolescents.

H3: Psychological resilience negatively mediates (i.e., partially suppresses) the positive relationship between negative life events and suicidal ideation among adolescents.

**Figure 1:**
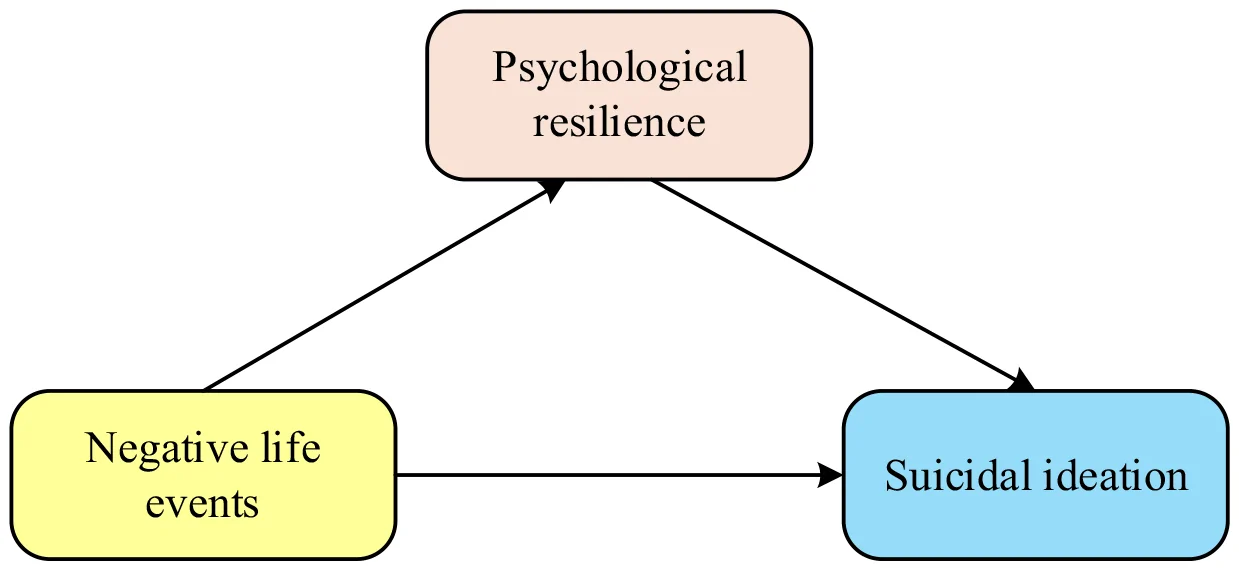
Hypothesis model of negative life events, psychological resilience, and suicidal ideation in adolescents.

The hypothetical theoretical model is shown in **Figure 1**.

### 3.2 Study population

This study conducted a secondary analysis using publicly accessible data from the Global School-based Student Health Survey (GSHS) – China, conducted in 2003 in four major cities (Beijing, Hangzhou, Wuhan, and Urumqi), jointly developed by the World Health Organization (WHO) and the Centers for Disease Control and Prevention (CDC). The GSHS is a school-based survey designed to assess health risk behaviors and protective factors among adolescents aged 13–17 years. It employs a standardized, self-administered questionnaire across ten core modules and has been implemented in over 100 low- and middle-income countries since 2003. The GSHS has demonstrated adequate test-retest reliability and cross-cultural validity across diverse settings. The China GSHS survey followed the standardized GSHS protocol with country-specific adaptations approved by WHO/CDC. The GSHS employs a two-stage cluster sampling design: schools are first randomly selected within defined geographic strata, followed by random selection of classrooms within selected schools. All students in the selected classrooms are invited to participate. The original data collection was conducted by trained survey teams commissioned by WHO/CDC following standardized protocols. The sample is predominantly urban, as the survey was administered in city-based schools.

The GSHS China dataset initially included 3,012 respondents from middle- and high-school students aged 13–17. From the full GSHS China 2003 dataset (N = 9,015 across four cities), 3,012 respondents aged 13—17 with complete or near-complete data on the variables of interest were initially identified. For this secondary analysis, we extracted adolescents who provided complete and valid information on all variables of interest: negative life events (bullying frequency, physical bullying victimization, physical fight, serious injury, food insecurity), psychological resilience–related indicators (parental support, peer support, school connectedness), suicidal ideation, and demographic variables (gender, age group, parental support level, peer support, food insecurity status).

**Figure 2:**
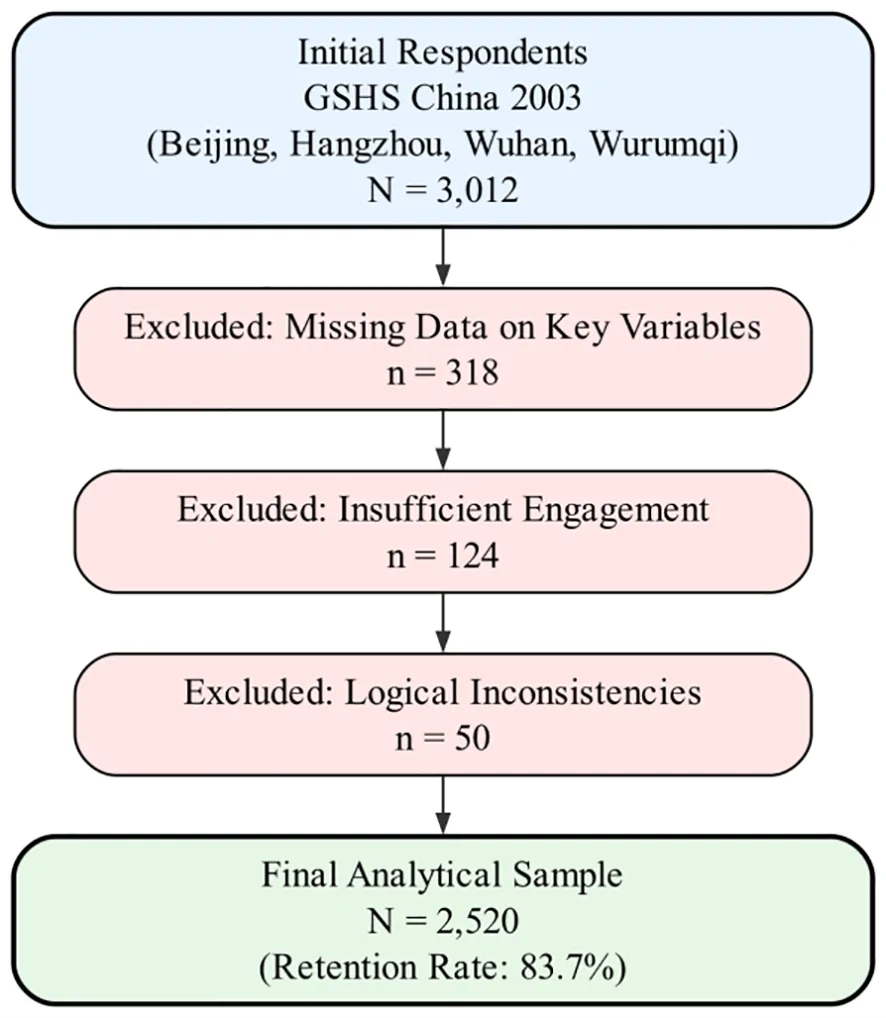
Participant flow diagram summarizing sample selection and attrition from the GSHS China 2003 survey.

Data-cleaning procedures excluded 492 cases due to: (a) missing responses on key variables (n=318), (b) systematic response patterns indicating insufficient engagement (e.g., straight-lining; n=124), and **(c)** logical inconsistencies (n=50). Comparison of excluded and retained cases showed no significant differences in age distribution (χ^2^ = 2.34, p=0.31) or gender ratio (χ^2^ = 1.89, p=0.17), suggesting minimal selection bias. A participant flow diagram summarizing sample selection and attrition is provided in **Figure 2**. The final analytical sample consisted of 2,520 adolescents (retention rate: 83.7%). As this study is a secondary analysis of an existing dataset, a formal *a priori* sample size calculation was not applicable. Among the 492 excluded cases, 318 were excluded due to missing responses on key variables. Within the age-eligible sample (13–17 years, N = 8,833), missingness rates for individual items were as follows: bullying frequency (Q20, 7.1%), physical bullying victimization (Q21, 7.3%), physical fight (Q14, 0.2%), serious injury (Q15, 19.2%, reflecting the skip pattern for uninjured respondents), food insecurity (Q6, 0.3%), parental understanding (Q53, 0.8%), parental monitoring (Q54, 0.9%), close friends (Q27, 0.7%), school connectedness (Q51, 1.0%), suicidal thoughts (Q25, 1.2%), and suicide plan (Q26, 1.1%). Apart from Q15 (which follows a conditional skip pattern), no single variable exceeded 8% missingness. The GSHS dataset is fully anonymized by the data providers, and no personally identifiable information is included. Age was categorized into two groups (13–14 and 15–17 years) based on developmental stages (early vs. middle adolescence), consistent with prior GSHS-based studies. **Table 1** presents the demographic characteristics of the final sample.

**Table 1:** Demographic characteristics of the sample.

Variable	Category	Number	Percentage (%)
Gender	Male	1,130	44.8
	Female	1,390	55.2
Age group	13–14 years	918	36.4
	15–17 years	1,602	63.6
Peer support (close friends)	≥ 1 friend	1,797	71.3
	0 friends	723	28.7
Food insecurity	Rare/Never	2,057	81.6
	Sometimes/Often	463	18.4
Parental support	High	1,467	58.2
	Low	1,053	41.8

Peer support was classified as having at least 1 close friend vs. 0 close friends. Food insecurity was classified as Rare/Never vs. Sometimes/Often based on the original GSHS response categories. Parental support was classified as high vs. low based on a median split of the composite score derived from two GSHS parental support items. Age group was classified as 13–14 years (early adolescence) vs. 15–17 years (middle adolescence) based on developmental stage.

### 3.3 Measurement tools

The Global School-based Student Health Survey (GSHS) incorporates standardized, internationally validated items designed to evaluate adolescents’ health risks, psychological well-being, and behavioral exposures. In this secondary analysis of the China GSHS dataset, the key constructs—negative life events, psychological resilience, and suicidal ideation—were operationalized using relevant survey items aligned with established WHO/CDC guidelines, ensuring comparability and reliability across global contexts. The exact survey items and variable codes for each construct are provided in **Supplementary Table 1**. Internal consistency was assessed using Cronbach’s alpha: negative life events (5 items, alpha = 0.81), psychological resilience (4 items, alpha = 0.74), and suicidal ideation (2 items, alpha = 0.78), all exceeding the acceptable threshold of 0.70 (**Table 2**). The construct validity of the measurement model was evaluated through confirmatory factor analysis (CFA) as reported in Section 4.6, where the model fit indices supported the adequacy of the latent structure.

**Table 2:** Reliability test results.

Scale	Number of items	Cronbach’s α
Negative life events	5	0.81
Psychological resilience	4	0.74
Suicidal ideation	2	0.78

#### 3.3.1 Negative life events

Negative life events were assessed through five GSHS items: bullying frequency, physical bullying victimization, physical fight, serious injury, and food insecurity. A composite score was derived by summing the five items, with possible score range 0-25. No reverse coding was applied. Higher values indicating greater cumulative exposure to negative life events, thereby reflecting the intensity of stressors commonly encountered by adolescents in school settings.

#### 3.3.2 Psychological resilience: proxy indicators and construct approximation

Psychological resilience was conceptualized as a set of protective factors that mitigate the impact of adversity, drawing from GSHS items related to supportive environments and relationships. Specifically, this encompassed four items: parental understanding, parental monitoring, close friends, and school connectedness. A composite score was derived by summing the four items (each coded 1—5), with possible score range 4—20. No reverse coding was applied. Higher composite scores reflect stronger protective resources, highlighting the role of familial, interpersonal, and institutional buffers in promoting adaptive coping among adolescents. It is important to acknowledge that contemporary resilience research conceptualizes resilience as a dynamic adaptive capacity encompassing both internal psychological resources (e.g., emotion regulation, cognitive flexibility) and external protective resources (e.g., social support, connectedness). The GSHS does not include a dedicated, validated psychological resilience scale; therefore, the available indicators—parental support, peer connectedness, number of close friends, and school belonging—serve as proxy measures representing key external protective resources that are consistently identified as core components within resilience frameworks. This approach of operationalizing resilience through available protective factors in large-scale health surveys follows established precedent, although it should be understood as a construct approximation rather than a direct, comprehensive measurement of the resilience process. Specifically, the present composite captures the protective resource dimension of resilience—the availability of supportive relationships and positive school environments—while necessarily omitting intrapsychic components such as coping self-efficacy and emotional regulation capacity that dedicated resilience scales would assess.

#### 3.3.3 Suicidal ideation

Suicidal ideation was evaluated using two GSHS items: suicidal thoughts and suicide plan. A composite score was derived by summing the two binary items (0/1 each), with possible score range 0—2. No reverse coding was applied. Higher values indicate more severe suicidal ideation, underscoring the need for early intervention in at-risk youth populations.

### 3.4 Data processing

All analyses were conducted using the publicly accessible GSHS China dataset. The GSHS complex sampling design employs sampling weights to account for non-response and selection probabilities; however, as the present study focuses on analytical associations within a mediation framework rather than population prevalence estimation, unweighted analyses were conducted. Missing data were handled using listwise deletion given the low proportion of missingness across the analytical variables. Standard data-cleaning procedures were applied, including removal of missing, contradictory, and incomplete cases. Subsequently, common method bias tests were conducted. Parametric tests (independent samples t-tests) were used for group comparisons, justified by the large sample size which ensures robustness to moderate normality violations per the Central Limit Theorem. Spearman’s correlation analysis was used for bivariate associations given the ordinal nature of the composite scores. Structural equation modeling was employed to analyze the mediating effect of psychological resilience between adolescents’ negative life events and suicidal ideation, using maximum likelihood estimation. GSHS items with ordinal response scales were treated as continuous variables, consistent with established practice for Likert-type items and given the robustness of maximum likelihood estimation to moderate violations of normality in large samples. The mediating effect was tested using 1,000 Bootstrap samples. All statistical analyses were conducted using SPSS 26.0 and AMOS 26.0. A p-value of<0.05 was considered statistically significant.

A mediation effect model was established with negative life events as the independent variable, suicidal ideation as the dependent variable, and psychological resilience as the mediating variable. The formula for calculating the mediation effect as shown in Equation 1 and 2:

(1) Indirect Effect=a×b

(2) $$\text{Total Effect}=c=c^{\prime }+\left(a\times b\right)$$

Where a represents the regression coefficient of negative life events on psychological resilience; b represents the regression coefficient of psychological resilience on suicidal ideation; c represents the total effect; and c’ represents the direct effect.

## 4 Result analysis and discussion

### 4.1 Common method bias test

This study used Harman’s single-factor test to assess common method bias. Specifically, the three key variables of negative life events, psychological resilience, and suicidal ideation were all included in the factor analysis to test the results of the unrotated factor analysis. The test results showed that this study involved 29 factors with characteristic root coefficients greater than 1, and the variance explained by the first common factor was approximately 2.18%. While this value appears low, it reflects the multidimensional nature of the constructs measured, with each composite measure comprising multiple distinct items from the GSHS questionnaire. The extraction of 29 factors with eigenvalues greater than 1 indicates that the items load onto their respective theoretical constructs rather than a single common factor. According to the testing criteria—that is, the number of factors with eigenvalues greater than 1 should be more than one, and the variance explained by the largest factor should be less than 40%—the testing results did not show any instances of only one factor being extracted or any factor having an abnormally high variance explained. This indicates that there was no severe common method bias effect among the variables studied in this paper.

### 4.2 Internal consistency

Internal consistency of the three composite measures was satisfactory (α = 0.74—0.81; **Table 2**). Negative life events comprised five items: bullying frequency, physical bullying victimization, physical fight, serious injury, and food insecurity (α = 0.81). Psychological resilience included four items: parental understanding, parental monitoring, close friends, and school connectedness (α = 0.74). Suicidal ideation consisted of two items: suicidal thoughts and suicide plan (α = 0.78).

### 4.3 Descriptive statistics

This study analyzed publicly available GSHS survey data on adolescents’ negative life events, psychological resilience, and suicidal ideation. The basic statistical information for each variable is presented in **Table 3**.

**Table 3:** Descriptive statistics for main variables.

Variable	Min	Max	Mean	SD
Negative life events (composite score)	0	25	8.2	4.8
Psychological resilience (composite score)	4	20	14.3	3.6
Suicidal ideation (composite score)	0	2	0.8	1.2

In this study, descriptive statistical analysis was employed to examine the overall profile of adolescents’ negative life events, psychological resilience, and suicidal ideation based on GSHS composite scores. As indicated in **Table 3**, the mean composite score for negative life events was 8.2 ± 4.8, for psychological resilience was 14.3 ± 3.6, and for suicidal ideation was 0.8 ± 1.2. Additional sociodemographic and lifestyle characteristics (physical activity and tobacco use) are presented in **Supplementary Table 2**. Item-level response frequencies for each construct are presented in **Supplementary Table 3**.

### 4.4 Demographic differences in suicidal ideation

As exploratory analyses, chi-square tests were conducted to examine demographic differences in suicidal ideation; interaction effects were not formally tested. Dichotomization of continuous or multi-level categorical variables was applied to facilitate subgroup comparisons and clinical interpretability, although this approach reduces statistical precision. **Table 4** presents the distribution of suicidal ideation by gender, age group, parental support, peer support, and food insecurity. The results showed significant differences across all examined demographic variables. Female adolescents reported a higher detection rate of suicidal ideation (31.4%) than males (24.6%). Younger students aged 13–14 also reported higher suicidal ideation rates than older adolescents. Students with low parental support, no close friends, and frequent experiences of food insecurity exhibited markedly higher rates of suicidal ideation. These findings align with established public health research, which identifies social support and basic living security as critical protective factors against youth suicidal thoughts and behaviors.

**Table 4:** Suicidal ideation detection rates by demographic characteristics.

Variable	Category	Detection rate (%)	χ^2^
Gender	Male	24.6	6.82*
	Female	31.4	
Age group	13–14	33.1	11.29**
	15–17	25.4	
Parental support	High	18.7	22.53***
	Low	38.9	
Peer support	≥ 1 friend	25.2	8.41**
	0 friends	36.7	
Food insecurity	Rare/Never	24.3	17.92***
	Sometimes/Often	41.8	

***p< 0.001, **p< 0.01, *p< 0.05, the same below.

To investigate the impact of gender differences on the aforementioned variables, students were divided into two groups based on gender, and normality tests were conducted on these variables. The results indicated that the psychological resilience variable conformed to normality only in the female group (p > 0.05), while the remaining variables showed mild skewness. Given the large sample size, parametric t-tests were deemed appropriate, as the Central Limit Theorem ensures robustness to moderate departures from normality, and no extreme outliers were detected. Therefore, gender was treated as the independent variable, with adolescent negative life events, psychological resilience, and suicidal ideation composite scores as dependent variables for t-tests (see **Table 5**). The results showed that males had significantly higher scores in psychological resilience (15.2 ± 3.8) compared to females (13.6 ± 3.4) (t = -3.124, p = 0.002< 0.01). Differences in suicidal ideation and negative life events by gender did not reach statistical significance.

**Table 5:** Gender differences analysis of students’ variables.

Variable male	Male Mean ± SD	Female Mean ± SD	t-value	p-value
Negative life events (composite score)	7.8 ± 4.5	8.5 ± 5.0	-1.234	0.218
Psychological resilience (composite score)	15.2 ± 3.8	13.6 ± 3.4	-3.124	0.002**
Suicidal ideation (composite score)	0.7 ± 1.1	0.9 ± 1.3	-0.789	0.43

**p < 0.01.

The results indicated that males had significantly higher psychological resilience composite scores (15.2 ± 3.8) compared to females (13.6 ± 3.4) (t = -3.124, p = 0.002< 0.01), suggesting that male adolescents may have stronger protective factors, including parental support, peer support, school connectedness, and social networks. However, no significant gender differences were found in negative life events or suicidal ideation composite scores.

To explore the impact of age group differences on the aforementioned variables, all students were classified into 13–14 years old and 15–17 years old groups, and normality tests were conducted. The results showed that the psychological resilience variable conformed to normality in both age groups (p > 0.05 in most cases), with mild skewness observed. Given the large sample size, parametric t-tests were appropriate, as the Central Limit Theorem ensures robustness to moderate departures from normality, and no extreme outliers were detected. Therefore, age group (13–14 vs. 15–17) was treated as the independent variable, with adolescent negative life events, psychological resilience, and suicidal ideation composite scores as dependent variables for t-tests (see **Table 6**). The results indicated that 13–14 years old students had significantly higher negative life events composite scores (9.5 ± 5.2) compared to 15–17 years old students (7.3 ± 4.4) (t = 4.567, p< 0.001). Differences in suicidal ideation across age groups reached statistical significance, with 13–14 years old students showing higher composite scores (1.1 ± 1.4) than 15–17 years old students (0.6 ± 1.0) (t = 2.456, p = 0.014< 0.05). Psychological resilience differences also reached significance, with 13–14 years old students having lower composite scores (13.5 ± 3.5) than 15–17 years old students (14.8 ± 3.6) (t = -2.123, p = 0.034< 0.05).

**Table 6:** Age group differences analysis of students’ variables.

Variable	13–14 years old Mean ± SD	15–17 years old Mean ± SD	t-value	p-value
Negative life events (composite score)	9.5 ± 5.2	7.3 ± 4.4	4.567	<0.001***
Psychological resilience (composite score)	13.5 ± 3.5	14.8 ± 3.6	-2.123	0.034*
Suicidal ideation (composite score)	1.1 ± 1.4	0.6 ± 1.0	2.456	0.014*

*p< 0.05; ***p< 0.001.

The age group differences analysis revealed that younger adolescents (13–14 years) reported significantly higher exposure to negative life events and suicidal ideation, along with lower psychological resilience, compared to older adolescents (15–17 years). These findings suggest that early adolescence represents a particularly vulnerable period, highlighting the importance of targeted interventions for this age group.

To examine the differences in variables based on parental support, all students were classified into high parental support and low parental support groups, and normality tests were conducted. The results showed that the psychological resilience variable conformed to normality in both groups (p > 0.05), while the remaining variables exhibited mild skewness. Therefore, parental support was treated as the independent variable, with adolescent negative life events (composite score), psychological resilience (composite score), and suicidal ideation (composite score) as dependent variables for t-tests (see **Table 7**). The results displayed that the negative life events composite score was significantly higher in low parental support (9.8 ± 5.4) compared to high parental support (7.1 ± 4.2) (t = 3.012, p = 0.003< 0.01). Psychological resilience composite score showed no significant difference (t = 1.456, p = 0.146 > 0.05). Suicidal ideation composite score was significantly higher in low parental support (1.2 ± 1.5) than in high parental support (0.5 ± 0.9) (t = -2.456, p = 0.014< 0.05).

**Table 7:** Parental support differences analysis of students’ variables.

Variable	High parental support Mean ± SD	Low parental support Mean ± SD	t-value	p-value
Negative life events (composite score)	7.1 ± 4.2	9.8 ± 5.4	3.012	0.003**
Psychological resilience (composite score)	14.5 ± 3.7	14.0 ± 3.5	1.456	0.146
Suicidal ideation (composite score)	0.5 ± 0.9	1.2 ± 1.5	-2.456	0.014*

*p < 0.05; **p < 0.01.

The results revealed that adolescents with low parental support reported significantly higher negative life events composite scores (9.8 ± 5.4) compared to those with high parental support (7.1 ± 4.2) (t = 3.012, p = 0.003< 0.01), including higher exposure to bullying, physical attacks, fights, injuries, and food insecurity. Adolescents with low parental support also showed significantly higher suicidal ideation composite scores (1.2 ± 1.5) than those with high parental support (0.5 ± 0.9) (t = -2.456, p = 0.014< 0.05), highlighting the critical protective role of parental support in reducing both adverse experiences and suicidal thoughts.

### 4.5 Correlation analysis between variables

Normality tests were conducted on the scores of the three variables, revealing that none conformed to normality (p< 0.05), though skewness was mild. Therefore, Spearman’s correlation analysis was employed to examine the relationships between adolescents’ negative life events, psychological resilience, and suicidal ideation (see **Table 8**).

**Table 8:** Correlations between negative life events, psychological resilience, and suicidal ideation.

Variable	Negative life events	Psychological resilience	Suicidal ideation
Negative Life Events	1	-0.512***	0.456***
Psychological Resilience	-0.512***	1	-0.623***
Suicidal Ideation	0.456***	-0.623***	1

***p< 0.001.

As shown in **Table 8**, the correlation coefficients between the variables all reached statistical significance (r = 0.456, p< 0.001), indicating a moderate positive correlation. Psychological resilience showed a negative correlation with both negative life events (r = -0.512***) and suicidal ideation (r = -0.623***), indicating a moderate to strong negative correlation. Overall, these findings underscore the protective role of psychological resilience against the adverse effects of negative life events on suicidal ideation.

### 4.6 Model fit assessment

Confirmatory factor analysis (CFA) was conducted to assess the model fit. The goodness-of-fit indices and their corresponding results are summarized in **Table 9**. In structural equation modelling (SEM), the following fit indices are commonly used to evaluate model adequacy, along with their computational formulas:

**Table 9:** Structural model fit indices.

Fit index	Value	Threshold	Assessment
χ^2^/df	2.134	1–3	Good
RMSEA	0.038	< 0.05	Excellent
CFI	0.962	> 0.90	Excellent
IFI	0.957	> 0.90	Excellent
TLI	0.948	> 0.90	Good

Root Mean Square Error of Approximation (RMSEA), as shown in Equation 3:

(3) $$RMSEA=\sqrt{\frac{\chi^{2}-df}{df\times \left(N-1\right)}}$$

Where *χ*^2^ is the chi-square value, *df* denotes degrees of freedom, and N*N* is the sample size.

Comparative Fit Index (CFI), as shown in Equation 4:

(4) $$CFI=1-\frac{max\left(\chi^{2}-df,0\right)}{max\left(\chi_{\text{baseline}}^{2}-df_{\text{baseline}},0\right)}$$

Where $\chi_{\text{baseline}}^{2}$ and $df_{\text{baseline}}$ represent the chi-square value and degrees of freedom of the baseline (independence) model, respectively.

Incremental Fit Index (IFI), as shown in Equation 5:

(5) $$IFI=\frac{\chi_{\text{baseline}}^{2}-\chi^{2}}{\chi_{\text{baseline}}^{2}-df}$$

Tucker–Lewis Index (TLI), as shown in Equation 6:

(6) $$TLI=\frac{\chi_{\text{baseline}}^{2}/df_{\text{baseline}}-\chi^{2}/df}{\chi_{\text{baseline}}^{2}/df_{\text{baseline}}-1}$$

The model fit in this study was evaluated using confirmatory factor analysis (CFA), with the results indicating a good overall fit. Specifically, the indices showed χ^2^/df = 2.134 (within the acceptable range of 1–3), RMSEA = 0.038 (less than 0.05), CFI and IFI exceeded 0.95, and TLI was 0.948, all exceeding the commonly recommended threshold of 0.90, indicating good model fit.

### 4.7 Analysis of the mediating effect of psychological resilience between adverse life events and suicidal ideation in adolescents

Structural equation modeling was employed to examine the mediating role of psychological resilience, with gender, age group, and food insecurity included as covariates in the model. The path coefficients were as follows: the effect of negative life events on psychological resilience (path a) was -0.252 (SE = 0.034, p< 0.001, β = -0.512), indicating that higher exposure to negative life events significantly reduced psychological resilience. The effect of psychological resilience on suicidal ideation (path b) was -0.272 (SE = 0.041, p< 0.001, β = -0.623), demonstrating that higher resilience was associated with lower suicidal ideation. The direct effect of negative life events on suicidal ideation (path c’) was 0.324 (SE = 0.038, p< 0.001, β = 0.330), which remained statistically significant after including the mediator, confirming partial mediation.

**Figure 3:**
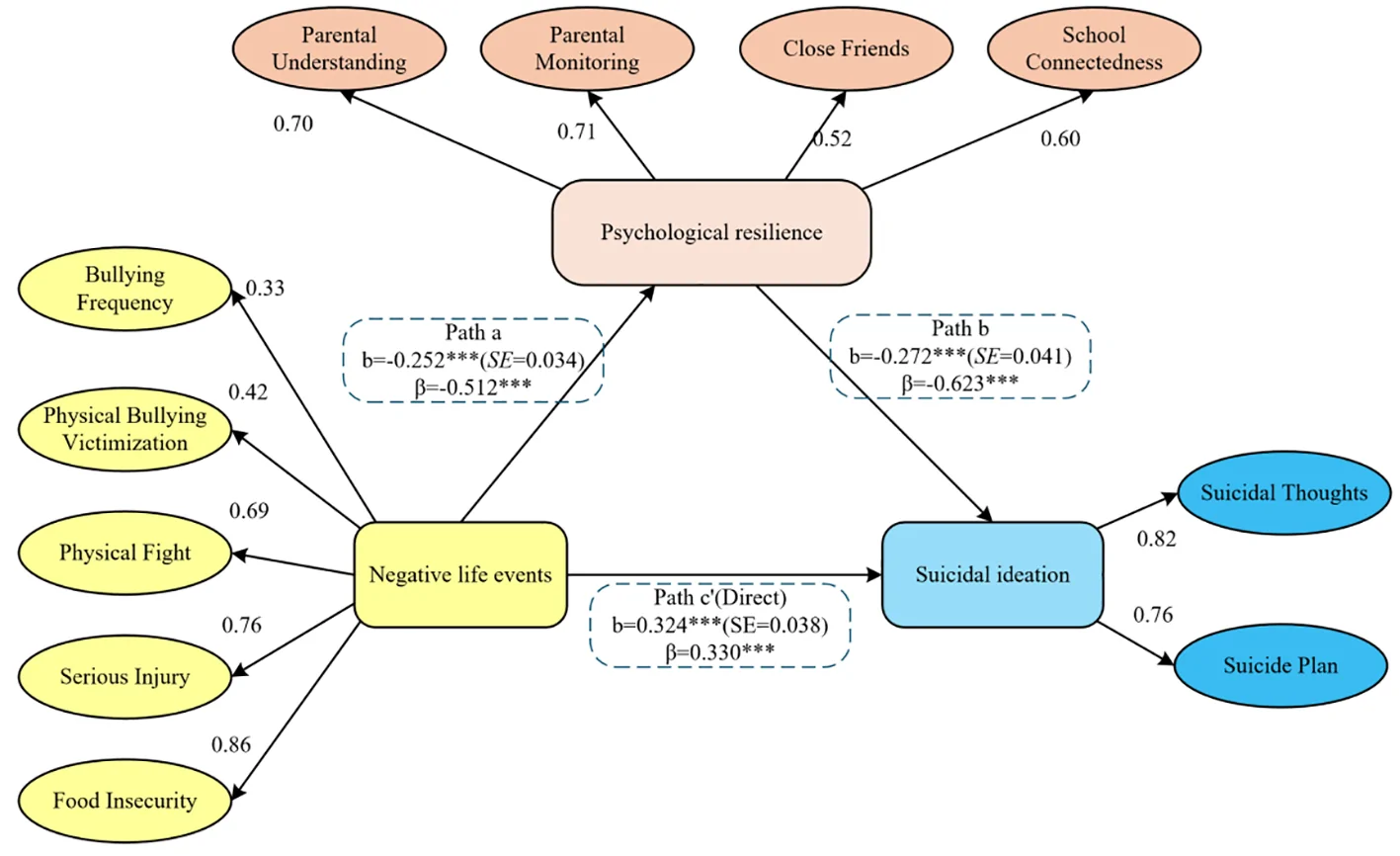
Mediating effect model of psychological resilience between negative life events and suicidal ideation in adolescents. Standardized path coefficients (β) are shown. Observed indicators are GSHS items: Q20 = bullying frequency, Q21 = physical bullying victimization, Q14 = physical fight, Q15 = serious injury, Q6 = food insecurity; Q53 = parental understanding, Q54 = parental monitoring, Q27 = close friends, Q51 = school connectedness; Q25 = suicidal thoughts, Q26 = suicide plan. Model fit: χ^2^/df = 2.134, RMSEA = .038, CFI = .962, TLI = .948. ***p<.001.

The total effect (c) of negative life events on suicidal ideation was 0.393 (p< 0.001). The indirect effect (mediation effect) through psychological resilience was calculated as a × b = (-0.252) × (-0.272) = 0.069, with a 95% bootstrap confidence interval (based on 1,000 samples) of [0.036, 0.527]. Since the CI does not include zero, the indirect effect is statistically significant. The proportion of the total effect mediated by psychological resilience was 17.56% (0.069/0.393), indicating that psychological resilience accounts for approximately one-sixth of the relationship between negative life events and suicidal ideation, while the majority (82.44%) represents the direct effect (see **Figure 3**).

## 5 Discussion

The present study examined the mediating role of psychological resilience in the relationship between negative life events and suicidal ideation among Chinese adolescents. This study is among the first to quantitatively estimate the mediating effect size of resilience-related protective resources in the NLE-SI relationship using a large sample of Chinese adolescents drawn from the GSHS. The findings provide empirical support for the theoretical framework suggesting that psychological resilience is associated with reduced adverse impact of negative life events on suicidal ideation.

The results revealed that negative life events were positively associated with suicidal ideation (r = 0.456, p< 0.001), consistent with previous research. Psychological resilience was negatively correlated with both negative life events (r = -0.512, p< 0.001) and suicidal ideation (r = -0.623, p< 0.001), supporting its role as a protective factor. The mediation analysis confirmed that psychological resilience partially mediated the relationship, with the mediation effect value of 0.069 accounting for 17.56% of the total effect (95% CI: 0.036—0.527).

A methodological consideration warrants attention when interpreting these findings. The resilience composite used in this study operationalizes protective resources—parental support, peer connectedness, and school belonging—that represent external, environmentally-mediated aspects of the resilience system. Contemporary resilience theory distinguishes between resilience as a dynamic adaptive process and the protective factors that support it. While the GSHS-based proxy indicators used here capture key protective resources consistently linked to resilient outcomes in adolescent research, they do not directly measure internal resilience capacities (e.g., coping flexibility, self-efficacy, emotional regulation). Consequently, the mediation effect (17.56% of the total effect) should be interpreted as reflecting the pathway through resilience-related protective resources rather than resilience per se. This distinction has implications for intervention design: programs targeting the external protective factors measured here (family support, peer networks, school climate) may operate through somewhat different mechanisms than those targeting internal resilience capacities.

The findings should also be interpreted within the Chinese sociocultural context. In Chinese society, which is characterized by collectivist values and strong family orientation, parental support and school connectedness may carry particular significance as resilience-related resources compared to individualistic Western contexts. The high academic pressure characteristic of the Chinese education system may also shape the types of negative life events most relevant to Chinese adolescents. Meanwhile, mental health stigma remains prevalent in Chinese culture, potentially influencing both the reporting of suicidal ideation and the utilization of available support resources. These cultural factors may affect the generalizability of the present findings and warrant cross-cultural comparative research.

Demographic analyses revealed important patterns. Suicidal ideation showed significant differences by gender, age group, parental support, peer support, and food insecurity. Female adolescents reported a higher detection rate of suicidal ideation (31.4%) than males (24.6%). Younger adolescents aged 13—14 reported significantly higher rates of suicidal ideation (33.1%) compared to older adolescents aged 15—17 (25.4%), as well as higher exposure to negative life events and lower psychological resilience. This pattern can be understood through developmental psychology frameworks: early adolescence represents a period of heightened neurobiological and social transitions, where prefrontal cortex development lags behind limbic system maturation. In contrast, older adolescents typically exhibit greater cognitive flexibility and more established coping strategies.

The partial mediation effect indicates that psychological resilience plays a modest but statistically significant role in the pathway from negative life events to suicidal ideation. The direct effect accounts for the majority (82.44%) of the relationship. This pattern is consistent with multi-pathway models of suicidal behavior, underscoring the complex, multifactorial nature of suicidal ideation.

## 6 Limitations

Several limitations should be considered. First, the cross-sectional design limits causal conclusions; cross-sectional mediation also assumes no residual confounding and temporal stability of constructs. Self-report measures may introduce recall and social desirability biases, and residual confounding from unmeasured variables cannot be ruled out, though demographic covariates were adjusted.

Second, the sample was drawn from four major Chinese cities (Beijing, Hangzhou, Wuhan, and Urumqi) and is predominantly urban; findings may not fully represent rural or out-of-school adolescents, who could face different risk profiles.

Third, psychological resilience was operationalized through GSHS proxy indicators (parental support, peer connectedness, school belonging) rather than a dedicated resilience scale, representing a construct approximation that captures external protective resources more directly than internal resilience capacities. Additionally, variable dichotomization may modestly reduce statistical precision, and parental support served both as a resilience component and a subgroup variable.

Future research employing longitudinal designs, more diverse samples, and validated multi-dimensional resilience scales would help clarify these mediating pathways.

## 7 Conclusion

This study examined the mediating role of psychological resilience in the relationship between negative life events and suicidal ideation among adolescents. The findings indicate that exposure to adverse experiences was associated with suicidal thoughts both directly and indirectly through resilience-related protective resources. Within the Chinese educational context, resilience-building programs could be integrated into existing school counseling services and moral education curricula, with targeted training for class teachers to identify at-risk students. At the family level, parent education programs emphasizing supportive parenting practices may enhance the protective role of parental support. At the community level, collaboration between schools and local mental health services could facilitate referral pathways for students requiring further psychological assessment.

## Data Availability

The original contributions presented in the study are included in the article/**Supplementary Material**. Further inquiries can be directed to the corresponding author.

## Supplementary material

Supplementary Table 1 GSHS China 2003 survey items, variable codes, and coding methods. Note. NLE, Negative Life Events; RES, Psychological Resilience; SI, Suicidal Ideation. Item wording is based on the standard GSHS core questionnaire modules; minor wording variations may exist in the China 2003 country-specific implementation. Q codes correspond to the GSHS China 2003 public-use dataset variable names. The Q21 variable in the GSHS China 2003 dataset uses an 8-value response scale; category labels reported here reflect the standard GSHS bullying module response format and may differ from the country-specific implementation. The Q51 item is from the GSHS Protective Factors module. NLE composite range: 5–37 (raw sum); RES composite range: 4–19; SI composite range: 0–2 (sum of two recoded binary items).

Supplementary Table 2 Physical activity and tobacco use characteristics of the sample. Note. Data are from GSHS China 2003 respondents aged 13–17 years (four cities: Beijing, Hangzhou, Wuhan, Urumqi). QN codes denote derived binary indicators (1, Yes, 2, No). qnminpag: met the WHO-recommended physical activity guideline of ≥60 minutes of moderate-to-vigorous physical activity per day on ≥5 days per week. QN32: includes chewing tobacco, snuff, cigars, pipes, and other smoked tobacco products. QN34: defined as exposure to tobacco smoke from others on ≥1 day in the past 7 days. Variables not available in the GSHS China 2003 dataset (school type, individual-level urban/rural residence, living arrangement with family) are acknowledged as data limitations in Section 6.

Supplementary Table 3 Item-level response frequencies for all construct items. Note. Data are from GSHS China 2003 respondents aged 13–17 years (four cities: Beijing, Hangzhou, Wuhan, Urumqi). NLE, Negative Life Events; RES, Psychological Resilience; SI, Suicidal Ideation. Percentages are based on valid (non-missing) responses for each item. For composite scoring, NLE and RES items were used as continuous variables with their original response scales; SI items were recoded so that Yes = 1 and No = 0. The Q21 variable in the GSHS China 2003 dataset uses an 8-value response scale; the first 7 categories are reported here, with the 8th category representing ≥12 instances. The high missingness rate for Q15 (19.3%) reflects the GSHS conditional skip pattern whereby respondents reporting no injuries were not asked follow-up injury items.
