# Supplementary material for: Psychological resilience partially mediates the relationship between negative life events and suicidal ideation in Chinese adolescents
Source: Front Psychiatry. 2026 Aug 20;17:1770147. doi: 10.3389/fpsyt.2026.1770147 (PMC13540038; doi:10.3389/fpsyt.2026.1770147)
Supplement: Supplementary file 1 [file SupplementaryFile1.docx]

**Supplementary Materials**

**Table S1. GSHS China 2003 Survey Items, Variable Codes, and Coding Methods**

| Construct | Variable (Q code) | GSHS Item Wording | Response Options | Coding in Present Study |
| --- | --- | --- | --- | --- |
| Negative Life Events (NLE) | Q20 | During the past 30 days, on how many days were you bullied? | 1 = 0 days; 2 = 1–2 days; 3 = 3–5 days; 4 = 6–9 days; 5 = 10–19 days; 6 = 20–29 days; 7 = All 30 days | Continuous (1–7); summed into NLE composite |
|  | Q21 | During the past 30 days, on how many days were you physically bullied (e.g., hit, kicked, pushed)? | 1 = 0 days; 2 = 1–2 days; 3 = 3–5 days; 4 = 6–9 days; 5 = 10–19 days; 6 = 20–29 days; 7 = All 30 days | Continuous (1–7); summed into NLE composite |
|  | Q14 | During the past 12 months, how many times were you in a physical fight? | 1 = 0 times; 2 = 1 time; 3 = 2–3 times; 4 = 4–5 times; 5 = 6–7 times; 6 = 8–9 times; 7 = 10–11 times; 8 = ≥12 times | Continuous (1–8); summed into NLE composite |
|  | Q15 | During the past 12 months, how many times were you seriously injured? | 1 = 0 times; 2 = 1 time; 3 = 2–3 times; 4 = 4–5 times; 5 = 6–7 times; 6 = 8–9 times; 7 = 10–11 times; 8 = ≥12 times | Continuous (1–8); summed into NLE composite |
|  | Q6 | During the past 30 days, how often did you go hungry because there was not enough food in your home? | 1 = Never; 2 = Rarely; 3 = Sometimes; 4 = Most of the time; 5 = Always | Continuous (1–5); summed into NLE composite |
| Psychological Resilience | Q53 | During the past 30 days, how often did your parents or guardians understand your problems and worries? | 1 = Never; 2 = Rarely; 3 = Sometimes; 4 = Most of the time; 5 = Always | Continuous (1–5); summed into RES composite |
|  | Q54 | During the past 30 days, how often did your parents or guardians really know what you were doing with your free time? | 1 = Never; 2 = Rarely; 3 = Sometimes; 4 = Most of the time; 5 = Always | Continuous (1–5); summed into RES composite |
|  | Q27 | How many close friends do you have? | 1 = 0; 2 = 1; 3 = 2; 4 = ≥3 | Continuous (1–4); summed into RES composite |
|  | Q51 | During the past 30 days, how often did you feel that you belonged at your school? | 1 = Never; 2 = Rarely; 3 = Sometimes; 4 = Most of the time; 5 = Always | Continuous (1–5); summed into RES composite |
| Suicidal Ideation (SI) | Q25 | During the past 12 months, did you ever seriously consider attempting suicide? | 1 = Yes; 2 = No | Recoded: Yes = 1, No = 0; summed into SI composite |
|  | Q26 | During the past 12 months, did you make a plan about how you would attempt suicide? | 1 = Yes; 2 = No | Recoded: Yes = 1, No = 0; summed into SI composite |

Note. NLE = Negative Life Events; RES = Psychological Resilience; SI = Suicidal Ideation. Item wording is based on the standard GSHS core questionnaire modules; minor wording variations may exist in the China 2003 country-specific implementation. Q codes correspond to the GSHS China 2003 public-use dataset variable names. The Q21 variable in the GSHS China 2003 dataset uses an 8-value response scale; category labels reported here reflect the standard GSHS bullying module response format and may differ from the country-specific implementation. The Q51 item is from the GSHS Protective Factors module. NLE composite range: 5–37 (raw sum); RES composite range: 4–19; SI composite range: 0–2 (sum of two recoded binary items).

**Table S2. Physical Activity and Tobacco Use Characteristics of the Sample**

| Variable | Category | n | % |
| --- | --- | --- | --- |
| Meets physical activity guideline (≥60 min/day on ≥5 days/week) | Yes | 6898 | 78.9 |
|  | No | 1844 | 21.1 |
|  | Missing | 121 | 1.4 |
| Attended physical education classes ≥3 days/week | Yes | 8112 | 92.2 |
|  | No | 684 | 7.8 |
|  | Missing | 67 | 0.8 |
| Walked or biked to school in past 7 days | Yes | 453 | 5.2 |
|  | No | 8337 | 94.8 |
|  | Missing | 73 | 0.8 |
| Spent ≥3 hours/day sitting (sedentary behavior) | Yes | 221 | 2.5 |
|  | No | 8556 | 97.5 |
|  | Missing | 86 | 1.0 |
| Any tobacco use in past 30 days | Yes | 553 | 6.5 |
|  | No | 7977 | 93.5 |
|  | Missing | 333 | 3.8 |
| Smoked cigarettes in past 30 days | Yes | 535 | 6.3 |
|  | No | 8022 | 93.7 |
|  | Missing | 306 | 3.5 |
| Used other tobacco products in past 30 days | Yes | 5479 | 62.2 |
|  | No | 3329 | 37.8 |
|  | Missing | 55 | 0.6 |
| Parent/guardian uses tobacco | Yes | 5475 | 62.1 |
|  | No | 3342 | 37.9 |
|  | Missing | 46 | 0.5 |
| Exposed to second-hand smoke in past 7 days | Yes | 1151 | 14.0 |
|  | No | 7062 | 86.0 |
|  | Missing | 650 | 7.3 |

Note. Data are from GSHS China 2003 respondents aged 13–17 years (four cities: Beijing, Hangzhou, Wuhan, Urumqi). QN codes denote derived binary indicators (1 = Yes, 2 = No). qnminpag: met the WHO-recommended physical activity guideline of ≥60 minutes of moderate-to-vigorous physical activity per day on ≥5 days per week. QN32: includes chewing tobacco, snuff, cigars, pipes, and other smoked tobacco products. QN34: defined as exposure to tobacco smoke from others on ≥1 day in the past 7 days. Variables not available in the GSHS China 2003 dataset (school type, individual-level urban/rural residence, living arrangement with family) are acknowledged as data limitations in Section 6.

**Table S3. Item-Level Response Frequencies for All Construct Items**

| Construct | Q Code | Item Description | Response Category | n | % |
| --- | --- | --- | --- | --- | --- |
| NLE | Q20 | Bullied in past 30 days | 0 days | 5900 | 71.6 |
|  |  |  | 1–2 days | 1354 | 16.4 |
|  |  |  | 3–5 days | 428 | 5.2 |
|  |  |  | 6–9 days | 183 | 2.2 |
|  |  |  | 10–19 days | 126 | 1.5 |
|  |  |  | 20–29 days | 59 | 0.7 |
|  |  |  | All 30 days | 187 | 2.3 |
|  |  |  | Missing | 626 | 7.1 |
| NLE | Q21 | Physically bullied in past 30 days | 0 days | 5896 | 71.8 |
|  |  |  | 1–2 days | 444 | 5.4 |
|  |  |  | 3–5 days | 81 | 1.0 |
|  |  |  | 6–9 days | 24 | 0.3 |
|  |  |  | 10–19 days | 249 | 3.0 |
|  |  |  | 20–29 days | 149 | 1.8 |
|  |  |  | All 30 days | 358 | 4.4 |
|  |  |  | Missing | 646 | 7.3 |
| NLE | Q14 | Physical fight in past 12 months | 0 times | 7198 | 81.4 |
|  |  |  | 1 time | 728 | 8.2 |
|  |  |  | 2–3 times | 530 | 6.0 |
|  |  |  | 4–5 times | 144 | 1.6 |
|  |  |  | 6–7 times | 51 | 0.6 |
|  |  |  | 8–9 times | 28 | 0.3 |
|  |  |  | 10–11 times | 22 | 0.2 |
|  |  |  | ≥12 times | 144 | 1.6 |
|  |  |  | Missing | 18 | 0.2 |
| NLE | Q15 | Seriously injured in past 12 months | 0 times | 5690 | 79.5 |
|  |  |  | 1 time | 1033 | 14.4 |
|  |  |  | 2–3 times | 322 | 4.5 |
|  |  |  | 4–5 times | 47 | 0.7 |
|  |  |  | 6–7 times | 23 | 0.3 |
|  |  |  | 8–9 times | 8 | 0.1 |
|  |  |  | 10–11 times | 3 | 0.0 |
|  |  |  | ≥12 times | 28 | 0.4 |
|  |  |  | Missing | 1709 | 19.3 |
| NLE | Q6 | Went hungry due to lack of food in past 30 days | Never | 4723 | 53.5 |
|  |  |  | Rarely | 2631 | 29.8 |
|  |  |  | Sometimes | 1282 | 14.5 |
|  |  |  | Most of the time | 154 | 1.7 |
|  |  |  | Always | 42 | 0.5 |
|  |  |  | Missing | 31 | 0.3 |
| RES | Q53 | Parents understood problems/worries in past 30 days | Never | 1572 | 17.9 |
|  |  |  | Rarely | 2136 | 24.3 |
|  |  |  | Sometimes | 1923 | 21.9 |
|  |  |  | Most of the time | 1962 | 22.3 |
|  |  |  | Always | 1201 | 13.7 |
|  |  |  | Missing | 69 | 0.8 |
| RES | Q54 | Parents knew about free-time activities in past 30 days | Never | 1131 | 12.9 |
|  |  |  | Rarely | 1911 | 21.8 |
|  |  |  | Sometimes | 1769 | 20.1 |
|  |  |  | Most of the time | 2219 | 25.3 |
|  |  |  | Always | 1752 | 19.9 |
|  |  |  | Missing | 81 | 0.9 |
| RES | Q27 | Number of close friends | 0 | 646 | 7.3 |
|  |  |  | 1 | 1037 | 11.8 |
|  |  |  | 2 | 1561 | 17.7 |
|  |  |  | ≥3 | 5558 | 63.1 |
|  |  |  | Missing | 61 | 0.7 |
| RES | Q51 | Felt a sense of belonging at school in past 30 days | Never | 724 | 8.3 |
|  |  |  | Rarely | 1621 | 18.5 |
|  |  |  | Sometimes | 2220 | 25.3 |
|  |  |  | Most of the time | 2602 | 29.7 |
|  |  |  | Always | 1607 | 18.3 |
|  |  |  | Missing | 89 | 1.0 |
| SI | Q25 | Seriously considered attempting suicide in past 12 months | Yes | 1513 | 17.3 |
|  |  |  | No | 7247 | 82.7 |
|  |  |  | Missing | 103 | 1.2 |
| SI | Q26 | Made a suicide plan in past 12 months | Yes | 713 | 8.1 |
|  |  |  | No | 8053 | 91.9 |
|  |  |  | Missing | 97 | 1.1 |

Note. Data are from GSHS China 2003 respondents aged 13–17 years (four cities: Beijing, Hangzhou, Wuhan, Urumqi). NLE = Negative Life Events; RES = Psychological Resilience; SI = Suicidal Ideation. Percentages are based on valid (non-missing) responses for each item. For composite scoring, NLE and RES items were used as continuous variables with their original response scales; SI items were recoded so that Yes = 1 and No = 0. The Q21 variable in the GSHS China 2003 dataset uses an 8-value response scale; the first 7 categories are reported here, with the 8th category representing ≥12 instances. The high missingness rate for Q15 (19.3%) reflects the GSHS conditional skip pattern whereby respondents reporting no injuries were not asked follow-up injury items.
